# The outcomes and prognostic factors in patients with osteosarcoma according to age: a Japanese nationwide study with focusing on the age differences

**DOI:** 10.1186/s12885-018-4487-2

**Published:** 2018-05-31

**Authors:** Yusuke Tsuda, Koichi Ogura, Yusuke Shinoda, Hiroshi Kobayashi, Sakae Tanaka, Akira Kawai

**Affiliations:** 1 Department of Orthopedic Surgery, University of Tokyo, 3-7-1 Hongo, Bunkyo-ku, Tokyo, Japan; 2 Department of Musculoskeletal Oncology and Rehabilitation Medicine, National Cancer Center Hospital, 5-1-1 Tsukiji, Chuo-ku, Tokyo, 104-0045 Japan

**Keywords:** Osteosarcoma, Elderly, Prognosis, Prognostic factor, Adjuvant chemotherapy

## Abstract

**Background:**

Few reports have described clinical features, prognosis and prognostic factors of osteosarcoma patients according to age.

**Methods:**

Using the Bone and Soft Tissue Tumor Registry in Japan, we identified 1043 osteosarcoma patients including 760 who were younger than 40 years, 173 aged between 41 and 64 years, and 110 patients older than 65 years. We extracted data on patient demographics and prognosis. Prognostic factors for patients older than 65 years or other age groups were analyzed.

**Results:**

Patients older than 65 years showed a significantly higher proportion of tumors arising in the trunk and with metastasis at diagnosis, and their 5-year disease-specific survival (DSS) rate was 32.7%. Multivariate analysis showed that the presence of metastasis at diagnosis [hazard ratio (HR): 3.04; 95% confidence interval (CI), 1.63–5.69; *P* < 0.001] and tumors > 16 cm in size (HR: 2.84 compared with < 8 cm; 95% CI, 1.16–6.97; *P* = 0.023) were significantly associated with worse DSS. The 5-year DSS was 39.1% in 80 patients older than 65 years without metastasis at diagnosis. Methotrexate was used in only 5.0% of these patients. Adjuvant chemotherapy was not significantly associated with better DSS (*P* = 0.323) in this generation and aged between 41 and 64 years (*P* = 0.566), although adjuvant chemotherapy yielded significantly better survival in patients younger than 40 years (*P* < 0.001).

**Conclusions:**

Analysis of this cohort of osteosarcoma patients revealed some unique clinical, therapeutic and prognostic features according to age groups in the largest cohort. Adjuvant chemotherapy was not associated with a better DSS in the group of patients aged between 41 and 64 years or older than 65 years.

## Background

Osteosarcoma is a rare malignant bone tumor with a predilection for adolescents and young adults. In elderly patients, osteosarcoma is often secondary to Paget’s disease or previous radiotherapy [1, 2]. However, approximately half of osteosarcomas in patients older than 60 years are primary tumors [3]. Because Paget’s disease of the bone and malignant bone tumor associated with Paget’s disease are uncommon in Japan [4–6], elderly osteosarcoma patients are relatively rare, and the age distribution of osteosarcoma has shown a single peak in the second decade [7, 8].

The proportion of the elderly population is growing rapidly worldwide, and this trend is particularly evident in Japan, where the elderly older than 65 years of age accounted for 25% of the population in 2013, being the highest in the world [9]. Accordingly, the number of elderly patients with primary osteosarcoma has been increasing [7]. As a result, the age distribution of osteosarcoma in Japan is now bimodal, with a peak in the seventh decade as well as in the second decade [7]. In other countries with aging populations, the absolute number of elderly patients with not only secondary but also primary osteosarcoma is expected to increase.

Advances in neoadjuvant chemotherapy including methotrexate (MTX), adriamycin (ADR) and cisplatin (CDDP) have improved the 5-year disease-specific survival (DSS) rate of osteosarcoma patients to more than 60% [10–14]. These data are not applicable to adult patients older than 40 years because all trials have included a younger population. Moreover, even in the largest study of osteosarcoma patients older than 65 years so far, only 43 cases were investigated [15]. Therefore, little is known about the clinical features and outcomes of osteosarcoma patients older than 65 years. However, studies focusing on this generation are crucial in order to develop an optimal treatment strategy because these patients often have unique problems such as a reduced performance status and several comorbidities [16]. In addition, the differences of treatment or prognosis in osteosarcoma among age groups have not been clear.

In the present study, we aimed to clarify the clinical features, outcomes and prognostic factors of osteosarcoma patients according to age groups using the Bone and Soft Tissue Tumor (BSTT) Registry, a nationwide organ-specific cancer registry of bone and soft tissue tumors in Japan. We also attempted to assess the effectiveness of adjuvant chemotherapy in the elderly generation.

## Methods

### Data source

The BSTT Registry is a nationwide organ-specific cancer registry for bone and soft tissue tumors in Japan. Details of the BSTT have been reported elsewhere [7, 17]. Detailed data for patients treated at the participating hospitals are collected annually in a clinician-oriented manner. The survey collects data in two sets. The first survey is conducted annually in May for patients treated between January 1 and December 31 of the previous year, and includes the following data for each patient: 1) basic data related to the patient: hospital, sex, age, date of diagnosis, status at the first visit, etc.; 2) information on the tumor: origin of the tumor (bone, soft tissue), histologic details (malignant or benign, and diagnosis), tumor location, the data required for TNM and Enneking staging (tumor size, nodal or distant metastasis, and histologic grade for malignant tumors.); 3) information on surgery: date of definitive surgery, type of surgery, reconstruction details, additional surgery for complications, etc.; and 4) information on treatments other than surgery: details of chemotherapy and radiotherapy. The second survey collects information on prognosis at 2, 5, and 10 years after the initial registration only for patients with bone and soft tissue sarcomas. It includes information on several outcome measures at the time of the latest follow-up, such as local recurrence, distant metastasis, oncologic outcome and limb salvage status. Use of the BSTT Registry for the purposes of clinical research was initiated in 2014 after approval from the Musculoskeletal Tumor Committee of the Japanese orthopaedic association (JOA). Approval for the present study was obtained from the Institutional Review Board of the JOA.

### Patients

Data were obtained from the BSTT Registry during 2006–2013. Only patients with high grade osteosarcoma were included in this study. We extracted data of this period because the BSTT Registry started from 2006 and the enough follow-up periods were required. For each patient, we extracted the following data: year of registration, sex, age, status at first visit, tumor size, location, histologic diagnosis, details of the treatment (surgical and non-surgical), and outcome at the last follow-up. We were unable to correct the data about pathologic fracture or response to preoperative chemotherapy because the BSTT Registry did not collect such information. In Japan, adjuvant chemotherapy have been administered before and after surgery for osteosarcoma patients generally.

### Statistical analyses

The primary endpoint for outcome was the occurrence of tumor-related death. DSS was defined as the period from the date of diagnosis until tumor-related death. Patients without tumor-related death, or patients who died due to other causes, were censored at the last follow-up. Metastasis-free survival (MFS) was defined as the time period from the date of diagnosis until occurrence of metastasis, or until the last follow-up for patients without metastasis. The DSS and MFS were estimated using the Kaplan-Meier method, and survival curves were compared using the log-rank test. The factors associated with survival were analyzed using the Cox proportional hazards model. The alpha level for statistical significance was set at a *P* value of 0.05. All statistical analyses were two-sided and conducted using IBM SPSS version 19.0 (IBM SPSS, Armonk, NY, USA).

## Results

### Patients characteristics and treatments

During 2006–2013, we identified the records of 1043 patients with high grade osteosarcoma treated at 96 hospitals in the BSTT Registry. Of these patients, 110 (12.4%) were older than 65 years (Table 1). The proportions of patients with tumors arising in the trunk (45.5%) and with metastasis at diagnosis (26.4%) were significantly higher (*P* < 0.001) in patients older than 65 years than in other generations. On the other hand, the proportions of patients who underwent definitive surgery (61.8%) and chemotherapy (50.0%) were significantly lower in patients older than 65 years.

**Table 1:** Clinical and treatment characteristics for the patients overall according to age group

		Overall		≤40		41–64		≥65		*p* value
		No. of patients	%	No. of patients	%	No. of patients	%	No. of patients	%	
Total		1043		760	70.1	173	17.5	110	12.4	
Sex										
Male		572	54.8	426	56.1	93	53.8	53	48.2	0.286
Female		471	45.2	334	43.9	80	46.2	57	51.8	
Age (years), mean [range]				18 [4–40]		53 [41–64]		73 [65–91]		
Tumor size (cm), mean [range]				10.1 [2.0–34.0]		10.1 [2.5–33.0]		10.2 [0.8–25.0]		0.961
≤ 8 cm		402	38.5	296	38.9	70	40.5	36	32.7	0.664
> 8 cm and ≤ 16 cm		517	49.6	373	49.1	82	47.4	62	56.4	
> 16 cm		89	8.5	65	8.6	15	8.7	9	8.2	
Unknown		35	3.4	26	3.4	6	3.4	3	2.7	
Tumor location										
Femur		516	49.4	394	51.8	74	42.8	48	43.6	< 0.001
Tibia		204	19.6	180	23.7	18	10.4	6	5.5	
Fibula		27	2.6	23	3.0	3	1.7	1	0.9	
Humerus		80	7.7	65	8.6	13	7.5	2	1.8	
Distal extremity		52	5.0	42	5.5	7	4.1	3	2.7	
Trunk		164	15.7	56	7.4	58	33.5	50	45.5	
Metastasis at diagnosis										
Yes		174	16.7	111	14.6	34	19.7	29	26.4	< 0.001
No		865	82.9	646	85.0	139	80.3	80	72.7	
Unknown		4	0.4	3	0.4	0	0	1	0.9	
Definitive surgery	Yes	921	88.3	715	94.1	138	79.8	68	61.8	< 0.001
Chemotherapy	Yes	954	91.5	746	98.2	153	88.4	55	50.0	< 0.001
	Setting of chemotherapy									
	Adjuvant	832	79.8	674	88.7	125	72.3	33	30.0	< 0.001
	Palliative	110	10.5	62	8.2	27	15.6	21	19.1	
	Unknown	12	1.2	10	1.3	1	0.6	1	0.9	
	Chemotherapeutic agent									
	ADR	876	84.0	695	91.4	141	81.5	40	36.4	< 0.001
	IFO	681	65.3	542	71.3	112	64.7	27	24.5	< 0.001
	CDDP	840	80.5	691	90.9	124	71.7	25	22.7	< 0.001
	MTX	654	62.7	598	78.7	51	29.5	5	4.5	< 0.001
Radiotherapy	Yes	163	15.6	72	9.5	47	27.2	44	40.0	< 0.001
	Setting of radiotherapy									
	Adjuvant	32	19.6	16	22.2	9	19.1	7	15.9	0.098
	Palliative	55	33.7	19	26.4	13	27.7	23	52.3	
	Definitive	70	42.9	34	47.2	24	51.1	12	27.3	
	Unknown	6	3.7	3	4.2	1	2.1	2	4.5	

*ADR* adriamycin, *IFO* ifosfamide, *CDDP* cisplatin, *MTX* methotrexate

We identified the records of 865 osteosarcoma patients without metastasis at diagnosis. The proportion of patients with tumors arising in the trunk (37.5%) was significantly higher in patients older than 65 years than in the other generations, whereas the proportions of patients who underwent definitive surgery (61.8%) and chemotherapy (46.3%) were significantly lower in this group. Among chemotherapeutic drugs, MTX and CDDP were used in only 5.0 and 25.0% of patients older than 65 years, respectively.

### Outcomes

**Fig. 1:**
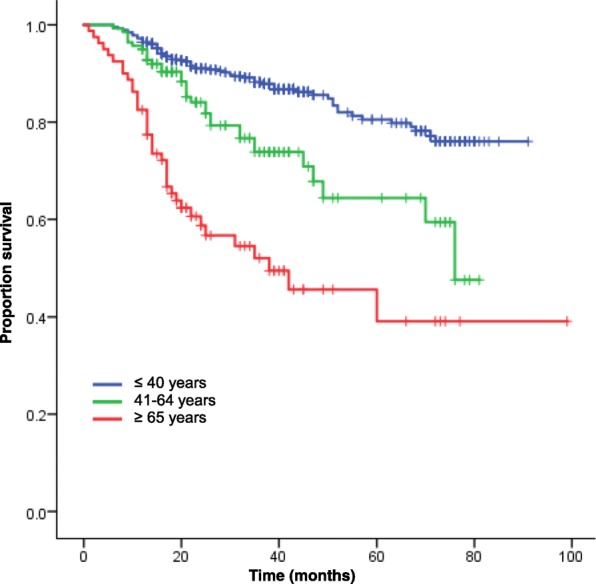
Kaplan-Meier curves of DSS for overall patients stratified by age

We analyzed the cumulative DSS for all patients according to age groups. The cumulative DSS at 3 and 5 years for patients older than 65 years was 42.8% and 32.7%, respectively, and these rates were the worst among the three age groups (*P* < 0.001, 5-year DSS: 56.9% for patients aged 40–65 years, 74.9% for patients younger than 40 years, Fig. 1).

We also analyzed the cumulative DSS for patients without metastasis at diagnosis according to age groups. The cumulative DSS at 3 and 5 years for 80 patients older than 65 years was 52.0 and 39.1%, respectively, and these rates were the worst among the three age groups (*P* < 0.001, 5-year DSS: 64.4% for patients aged 40–65 years, 80.5% for patients younger than 40 years).

### Prognostic factors

Table 2 shows the multivariate hazard ratios (HRs) obtained from the Cox models for DSS in osteosarcoma patients according to age groups. In the patients older than 65 years, univariate analysis showed that the presence of metastasis at diagnosis (*P* < 0.001), a tumor arising in the trunk (*P* = 0.007), a larger tumor (> 16 cm, *P* = 0.003) and lack of definitive surgery (*P* < 0.001) were significantly associated with poorer DSS. Multivariate analysis showed that the presence of metastasis at diagnosis [HR: 3.04; 95% CI (confidence interval), 1.63–5.69; *P* < 0.001] and a tumor > 16 cm in size (HR: 2.84 compared with < 8 cm; 95% CI, 1.16–6.97; *P* = 0.023) were significantly associated with poorer DSS in this age group.

**Table 2:** Multivariate analysis of prognostic factors for disease-specific survival of osteosarcoma patients according to age group

	≤40			41–64			≥65		
	No. of patients	Hazard ratio (CI)	*p* value	No. of patients	Hazard ratio (CI)	*p* value	No. of patients	Hazard ratio (CI)	*p* value
Total	760			173			110		
Sex									
Male	426	Reference		93	Reference		53	Reference	
Female	334	0.93 (0.65–1.32)	0.684	80	0.75 (0.42–1.33)	0.318	57	1.06 (0.63–1.78)	0.821
Metastasis at diagnosis									
No	646	Reference		139	Reference		80	Reference	
Yes	111	3.42 (2.27–5.15)	< 0.001	34	3.04 (1.63–5.69)	< 0.001	30	3.04 (1.63–5.69)	< 0.001
Tumor location									
Extremity	704	Reference		115	Reference		60	Reference	
Trunk	56	1.47 (0.83–2.60)	0.185	58	1.64 (0.80–3.36)	0.179	50	1.01 (0.53–1.93)	0.969
Tumor size									
≤ 8 cm	296	Reference		70	Reference		36	Reference	
> 8 cm and ≤ 16 cm	373	1.72 (1.13–2.61)	0.011	82	0.91 (0.50–1.68)	0.770	62	1.03 (0.58–1.82)	0.927
> 16 cm	65	2.11 (1.13–3.93)	0.019	15	1.50 (0.56–3.96)	0.429	9	2.84 (1.16–6.97)	0.023
Definitive surgery									
Yes	45	Reference		35	Reference		68	Reference	
No	715	3.74 (2.03–5.96)	< 0.001	138	1.01 (0.45–2.29)	0.982	42	1.74 (0.86–3.45)	0.124

*DSS* disease-specific survival, *CI* confidence interval

We also performed the univariate and multivariate analysis for DSS in osteosarcoma patients without metastasis at diagnosis. In patients older than 65 years, multivariate analysis showed that patients who underwent amputation (HR: 3.24; 95% CI, 1.12–9.36; *P* = 0.030) had significantly poorer DSS than those who underwent limb salvage surgery.

### Adjuvant chemotherapy in the various age groups

**Fig. 2:**
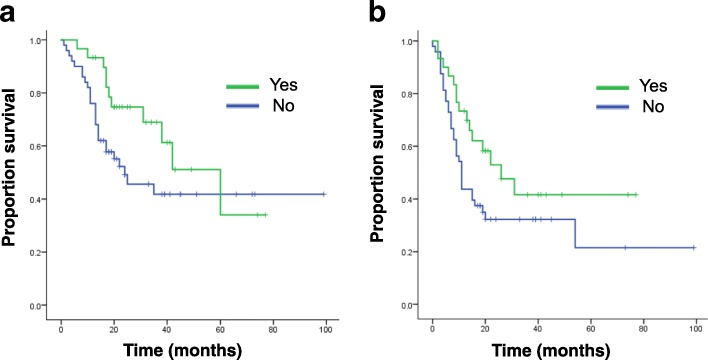
**a** Kaplan-Meier curves of DSS for patients older than 65 years without metastasis at diagnosis stratified by adjuvant chemotherapy. **b**. Kaplan-Meier curves of MFS for patients older than 65 years without metastasis at diagnosis stratified by adjuvant chemotherapy

Advances in neoadjuvant chemotherapy for osteosarcoma have improved the 5-year DSS to more than 60% in patients younger than 40 years [11–15]. In our study, adjuvant chemotherapy was not significantly associated with better DSS in patients older than 65 years without metastasis at diagnosis in the multivariate analysis (*P* = 0.323). The survival curves for DSS and MFS stratified by adjuvant chemotherapy also showed no statistically significant difference in this age group (Fig. 2a, b*, P* = 0.100 for DSS, *P* = 0.071 for MFS).

Multivariate analyses were conducted for other age groups (Aged 41–64 years or younger than 40 years). They showed that adjuvant chemotherapy was significantly associated with better outcome in patients younger than 40 years (*P* < 0.001), and not associated for those aged 41–64 years (*P* = 0.566).

## Discussion

In the present study, we analyzed 1043 patients with osteosarcoma including 110 patients who were older than 65 years. The patients older than 65 years showed significantly higher rates of tumors arising in the trunk and presence of metastasis at diagnosis. They less frequently received adjuvant chemotherapy, and key chemotherapeutic drugs, especially for MTX and CDDP, were used for only a few patients. The 5-year DSS was 32.7%, which was the worst among the three age groups. Larger tumors and the presence of metastasis at diagnosis were associated with poorer DSS in these older patients. Adjuvant chemotherapy was not significantly associated with a better DSS in patients older than 65 years without metastasis at diagnosis (*P* = 0.323). Our data demonstrated the difference of treatment, prognosis and prognostic factors according to age groups.

In recent years, the outcomes for osteosarcoma have improved due to advances in neoadjuvant chemotherapy [10–14]. Our data showed that the 5-year DSS for patients younger than 40 years was 74.9%, whereas that for patients older than 65 years was still quite poor (32.7%), and the worst among the three age groups. Similarly, Longhi et al. reported that the 5-year overall survival of elderly patients with osteosarcoma was 22% [15]. From our study, the poor outcomes for such elderly patients were likely due to the high proportion of tumors arising in the trunk or the presence of metastasis at diagnosis.

To improve treatment outcomes for this elderly generation, early diagnosis and treatment are needed because the presence of metastasis at diagnosis and a larger tumor size were identified as factors having a negative impact on DSS. In general, osteosarcoma has been recognized as a disease of adolescents and young adults. We may need to change our recognition and become more aware of osteosarcoma in the elderly to achieve earlier diagnosis and treatment. We also believe that development of viable and effective standardized chemotherapy or some form of novel therapy applicable to elderly patients is needed, because the present study revealed that key drugs such as MTX or CDDP could not be used in many cases.

**Fig. 3:**
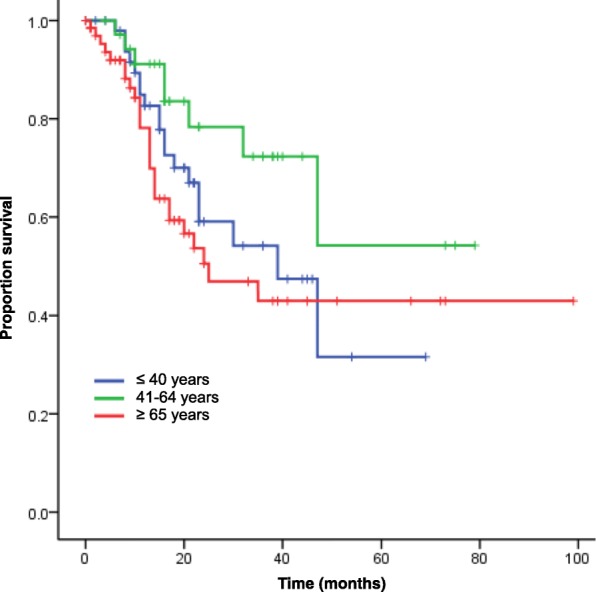
Kaplan-Meier curves of DSS for patients with localized tumors not receiving adjuvant chemotherapy stratified by age

Adjuvant chemotherapy is a standard option for young osteosarcoma patients with localized tumors [10–14]. International prospective studies have shown that a combination of MTX, CDDP and ADR is beneficial [11, 14]. However, those studies did not include patients older than 40 years. Our present study showed that addition of adjuvant chemotherapy in patients older than 65 years did not lead to improvement of either DSS and MFS. The limited range of drugs available for elderly patients, or dose limitations due to comorbidities or age-related organ dysfunction, probably accounted for our results. Another explanation is that osteosarcoma in older patients has a different biological nature and is more resistant to chemotherapy than that in younger patients. Recently, osteosarcoma in the elderly (average age 65 years) was reported to have unique genetic alterations, as represented by *H3F3A* mutation in addition to *TP53* or *LSAMP* mutation, and showed distinct DNA methylation profiles [18]. The differences in survival data among age groups for patients with localized tumors not receiving adjuvant chemotherapy may reflect this biological difference (5-year DSS: 41.8% for patients older than 65 years, 53.5% for patients aged 40–65 years, 31.4% for patients younger than 40 years, Fig. 3). Some previous retrospective studies have reported the results of adjuvant chemotherapy for osteosarcoma patients older than 40 years [5, 6, 15, 19–21]. In those studies, the effectiveness of adjuvant chemotherapy was controversial [5, 6, 15, 19–21]. Larger studies based on international collaboration or basic research to explore the biological differences in osteosarcoma are required in order to clarify the reasons for the variations in effectiveness of adjuvant chemotherapy.

Our study had several limitations. First, we were unable to detect secondary osteosarcoma since the BSTT Registry did not collect such information. However, Paget’s disease and malignant bone tumor associated with Paget’s disease are quite rare in Japan [4–6]. Therefore, we expect that most of the patients would have had primary osteosarcoma. Second, we were unable to control for several potentially important clinical parameters that may have affected the survival rate, such as pathologic fracture or response to preoperative chemotherapy, since the BSTT Registry did not collect such information. Third, we were unable to exclude the possibility of duplicate reporting if a patient had received care at more than one hospital. Because the patient data were de-identified before the participating hospitals submitted their data to the registry, in accordance with Japanese ethical guidelines, there was no way of excluding such duplicated cases. Finally, participation in the registry is not mandatory for non-JOA-certified hospitals. Although we expect that most osteosarcoma cases would be treated at specialist centers certified by the JOA, the possibility remains that some would have been treated at non-specialist hospitals due to patient preference or other reasons.

## Conclusions

Patients older than 65 years showed a significantly higher proportion of tumors arising in the trunk and with metastasis at diagnosis, and their 5-year DSS rate was the worst among three age groups. Adjuvant chemotherapy was not associated with a better survival in the group of patients older than 65 years or aged between 41 and 64 years. Our study emphasized the differences among age groups.

## Abbreviations

ADR: Adriamycin
BSTT: Bone and Soft Tissue Tumor
CDDP: Cisplatin
CI: Confidence interval
DSS: Disease-specific survival
HR: Hazard ratio
JOA: Japanese orthopaedic association
MFS: Metastasis-free survival
MTX: Methotrexate
